# Metabolic regulation of histone acetylation by ACLY supports MDR1 expression in colorectal cancer and highlights a targetable vulnerability

**DOI:** 10.1016/j.neo.2026.101314

**Published:** 2026-04-30

**Authors:** Ana García-Bautista, Aiora Cenigaonandia-Campillo, Anxo Rio-Vilariño, Lara Sanz-Criado, Marta Selva-Giménez, Raquel Perez-Antolín, Arancha Cebrián, Laura García-García, María Jesús Fernández-Aceñero, Natalia Baños-Herraiz, Lorena Mozas-Vivar, Estrella Núñez-Delicado, Jesús García-Foncillas, Óscar Aguilera

**Affiliations:** a Translational Oncology Division, OncohealthInstitute, IIS-Fundación Jimenez Diaz-UAM (Madrid), Spain; b Universidad Autónoma de Madrid. Facultad de Biología. Spain; c Hospital Clínico San Carlos (HCSC), (Madrid), Spain; d Preclinical programe START Madrid-FJD Hospital fundación Jiménez Díaz (Madrid) Spain; e Universidad Católica de Murcia (UCAM) Campus de losJerónimos 135, 30107 Guadalupe, Murcia, Spain; f Molecular Recognition and Encapsulation Research Group (REM), Health Sciences Department, Universidad Católica de Murcia (UCAM) Campus de losJerónimos 135, 30107 Guadalupe, Murcia, Spain

**Keywords:** Cancer, KRAS, MDR-1, ACLY, Ascorbate, Chemoresistance, Metabolism, Epigenetic

## Abstract

Chemoresistance remains a major cause of treatment failure in colorectal cancer (CRC), yet the metabolic mechanisms sustaining efflux-mediated drug resistance are not fully defined. Here, we identify ATP-citrate lyase (ACLY) as a metabolic regulator linking citrate-dependent acetyl-CoA production to epigenetic control of MDR1/ABCB1 expression.

Using genetic and pharmacologic approaches, we show that ACLY catalytic activity contributes to the maintenance of histone acetylation at H3K9 and H4K16 and supports MDR1 transcription in CRC cells. Consistently, ACLY overexpression enhances, whereas its inhibition reduces, MDR1 expression and associated resistance-related transcriptional programs. In human CRC specimens, ACLY and MDR1 levels positively correlate, with a stronger association observed in advanced-stage tumors, supporting clinical relevance of this metabolic–epigenetic axis.

Metabolic tracing with ^13^C-glucose suggests that perturbation of citrate flux influences ACLY-associated pathways and acetyl-CoA availability. In this context, vitamin C treatment reduces citrate-derived acetyl-CoA and ACLY phosphorylation and is associated with global histone deacetylation and decreased MDR1 expression *in vitro* and in KRAS-mutant patient-derived xenografts.

Together, these findings highlight ACLY-dependent acetyl-CoA production as a potential metabolic vulnerability linked to epigenetic regulation of drug efflux programs in CRC. Targeting this metabolic-chromatin axis may represent a strategy to modulate MDR1-associated chemoresistance.

## Highlights

• ACLY links metabolism to MDR1 expression in KRAS-mutant CRC.
• ACLY regulates MDR1 through acetyl-CoA–dependent chromatin control.
• Vitamin C disrupts citrate–ACLY metabolism and reduces MDR1 levels.
• ROS partially contribute to vitamin C–induced MDR1 downregulation.
• Vitamin C suppresses tumor growth and MDR1 in CRC PDX models.

## Graphical abstract

**Figure:**
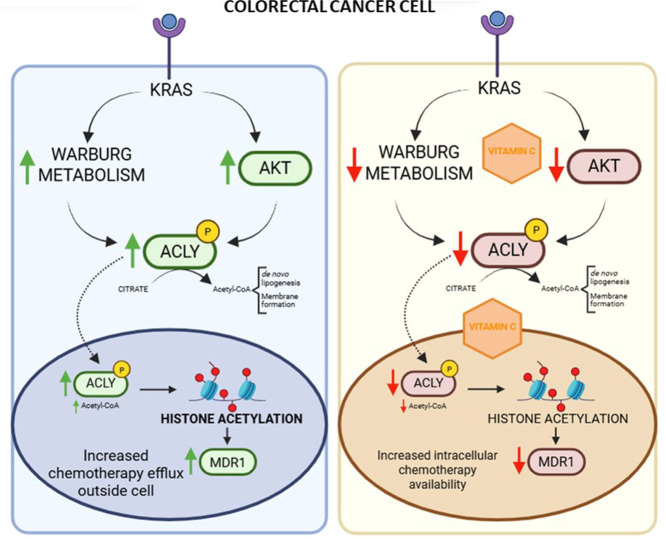

## Introduction

Chemoresistance remains a major cause of treatment failure in colorectal cancer (CRC), particularly in advanced disease where therapeutic options are limited and survival outcomes remain poor [1]. Molecular stratification based on RAS/RAF mutational status has improved therapeutic decision-making; however, KRAS-mutant tumours continue to exhibit limited response to targeted therapies and chemotherapy [2,3]. Despite recent advances, including the development of mutant-selective KRAS inhibitors [4,5] and KRAS-directed degraders [6], clinical efficacy is frequently compromised by intrinsic and adaptive resistance mechanisms.

Among these, ATP-binding cassette (ABC) transporters play a central role by limiting intracellular drug accumulation. ABCB1 (also known as MDR1 or P-glycoprotein) is one of the most extensively characterized efflux pumps and is frequently upregulated in CRC, contributing to resistance against multiple chemotherapeutic agents [7]. MDR1 expression is dynamically regulated and can be induced upon treatment, reinforcing acquired resistance phenotypes [[8], [9], [10], [11], [12]]. Although several strategies have attempted to inhibit MDR1 function or expression, including direct transporter inhibition or transcriptional targeting, clinical translation has been limited by toxicity, lack of specificity, and compensatory mechanisms [11,13,14]. These limitations underscore the need to identify upstream regulatory pathways that sustain MDR1 expression.

Accumulating evidence indicates that metabolic reprogramming is closely linked to transcriptional adaptation in cancer cells. In particular, lipid metabolism has been implicated in multidrug resistance, not only by shaping membrane composition and transporter activity but also by influencing signaling and transcriptional programs [[15], [16], [17]]. However, the mechanisms through which metabolic pathways directly regulate efflux transporter expression remain incompletely understood.

ATP-citrate lyase (ACLY) occupies a central position at the interface between metabolism and gene regulation. As a rate-limiting enzyme in de novo lipogenesis, ACLY generates nucleocytosolic acetyl-CoA from citrate, providing a critical substrate for both lipid biosynthesis and histone acetylation [[18], [19], [20]]. Through this function, ACLY links nutrient availability to chromatin remodeling and transcriptional control. Increased ACLY expression and activity have been associated with tumour progression and metabolic adaptation in CRC [18,19], and ACLY-dependent acetyl-CoA production has been implicated in chromatin regulation and DNA repair processes [[20], [21], [22]]. However, whether ACLY-driven acetyl-CoA availability directly regulates MDR1 transcription in CRC remains unclear. Moreover, alterations in lipid metabolism pathways have been shown to influence colorectal cancer progression, further supporting a role for metabolic processes in shaping tumor behavior [23].

Given that ACLY activity depends on citrate availability and metabolic flux through the tricarboxylic acid cycle, perturbation of upstream metabolic pathways may represent a strategy to modulate ACLY-dependent chromatin states. In this context, pharmacological vitamin C has been shown to impact cancer cell metabolism and epigenetic regulation, particularly in KRAS-mutant settings as previously reported [24]. Beyond its established roles in redox biology, vitamin C can influence central carbon metabolism and chromatin-associated processes, including pathways linked to acetyl-CoA availability. However, whether these effects intersect with ACLY-dependent transcriptional regulation remains unclear.

In the present study, we investigate the role of ACLY as a metabolic regulator of MDR1 expression in CRC. We demonstrate that ACLY catalytic activity sustains histone acetylation at H3K9 and H4K16 and maintains a chromatin environment permissive for MDR1 transcription. Furthermore, we show that metabolic perturbation using vitamin C disrupts citrate-dependent ACLY activity, reduces acetyl-CoA availability, and suppresses MDR1 expression *in vitro* and in patient-derived models. Together, our findings define a metabolic–epigenetic axis linking lipid metabolism to drug efflux programs and identify ACLY-dependent acetyl-CoA production as a potential therapeutic vulnerability in colorectal cancer.

## Methods

### Cell lines and culture conditions

Human colorectal cancer cell lines SW480 (KRASG12V) and DLD1 (KRASG13D) were obtained from the American Type Culture Collection (ATCC) and authenticated by short tandem repeat profiling. These models were selected as representative KRAS-mutant CRC systems. Cells were maintained in Dulbecco’s modified Eagle’s medium (DMEM) supplemented with 10% fetal bovine serum, 25 mM HEPES, and 100 U/mL penicillin at 37°C in a humidified atmosphere containing 5% CO₂. Mycoplasma contamination was routinely monitored every 30 days.

### Pharmacological treatments

Cells were seeded at 1–2 × 10⁶ cells and allowed to adhere overnight prior to treatment. The ACLY inhibitor BMS-303141 (MedChemExpress; HY-16107) was dissolved in DMSO (10 mM stock) and used at 50 μM for 48 h. Vorinostat (MedChemExpress; HY-10221) was dissolved in DMSO and used at 0.5 μM (SW480) or 3.5 μM (DLD1) for 48 h. Sodium ascorbate (Sigma-Aldrich; A7632) was freshly prepared in sterile water (100 mM stock) and used at 5 mM for 6 h unless otherwise indicated. Vehicle-treated cells served as controls.

### Generation of ACLY-overexpressing cell lines

The pLV[Exp]-Hygro-CMV-hACLY construct (NM_001303274.1; VectorBuilder) and corresponding empty vector controls were amplified in E. coli and purified using a QIAGEN Plasmid Maxi Kit. Lentiviral particles were generated in HEK293T cells using polyethyleneimine (PEI) transfection with packaging plasmids (pVSV-G, RRE, RRV). Viral supernatants were collected 48 h post-transfection, filtered (0.44 μm), supplemented with polybrene (10 μg/mL), and used to transduce target cells. Stable populations were selected using hygromycin for 72 h.

### Western blot analysis

Cells were lysed in RIPA buffer supplemented with protease and phosphatase inhibitors. Protein concentration was determined using a BCA assay. Equal amounts of protein (20 μg) were separated by SDS–PAGE and transferred to nitrocellulose membranes. Membranes were blocked in 5% milk and incubated with primary antibodies against ACLY, phospho-ACLY (Ser455), MDR1, acetyl-H3K9, acetyl-H4K16, and β-actin, followed by HRP-conjugated secondary antibodies. Signals were detected by chemiluminescence and quantified by densitometry.

### Immunohistochemistry

Formalin-fixed, paraffin-embedded sections were deparaffinized and subjected to antigen retrieval in low-pH buffer at 95°C for 20 min. Endogenous peroxidase activity was blocked prior to overnight incubation with primary antibodies. Detection was performed using HRP-conjugated polymer systems (EnVision) and diaminobenzidine. Slides were counterstained with hematoxylin. Protein expression was quantified using H-score methodology.

### Quantitative real-time PCR

Total RNA was extracted using the NucleoSpin RNA kit and reverse-transcribed from 200 ng RNA using the High Capacity cDNA Reverse Transcription Kit. Quantitative PCR was performed using TaqMan assays on a 7500 Fast Real-Time PCR System. Gene expression levels were normalized to ACTB using the ΔΔCt method. All reactions were performed in triplicate.

### Metabolite analysis and ^13^C-glucose tracing

For metabolic tracing experiments, 2.5 × 10⁵ cells were cultured in DMEM containing 5.5 mM glucose and 2 mM glutamine without pyruvate. Four hours prior to harvest, medium was replaced with DMEM containing 5.5 mM U-13C-glucose (or U-12C-glucose as control) with or without 5 mM ascorbate. Metabolites were extracted using 80% methanol containing d27-myristic acid as an internal standard. Samples were analyzed by LC–MS (Q Exactive Orbitrap) in negative ion mode. Data were processed using EL-MAVEN with natural isotope correction (POLLY), and metabolite abundance was normalized to protein content. Pyruvate was analyzed by MALDI-MS and processed using MMASS, as previously described [25].

### Proteomic analysis

Protein extracts were acetone-precipitated, digested using the PreOmicsiST kit, and analyzed using an Evosep One system coupled to a timsTOF Pro 2 mass spectrometer operating in diaPASEF mode. Data were acquired across a 400–1200 m/z range. Raw files were processed using DIA-NN (v1.8.126) [26] with a human spectral library, applying a 1% false discovery rate. Differential protein expression analysis was performed using TraianProt [27] and limma [28], and functional enrichment analysis was conducted using clusterProfiler and KEGG pathways [29,30].

### Tumour microarray analysis

Formalin-fixed paraffin-embedded tumour samples from 307 stage II and 112 stage IV CRC patients treated at Hospital Universitario Clínico San Carlos (Madrid, Spain) were included. Representative tumour regions were selected by a pathologist, and triplicate 0.6-mm cores were assembled using a TMA workstation (Beecher Instruments). ACLY and MDR1 expression levels were evaluated by immunohistochemistry and quantified using H-score analysis.

### Public dataset analysis

Transcriptomic data for ACLY and MDR1 in colorectal cancer and normal tissues were analyzed using GEPIA2, integrating TCGA and GTEx datasets. Correlation analyses were performed using publicly available CRC datasets.

### Patient-derived xenograft models

KRAS-mutant CRC PDX models were established by subcutaneous implantation of 2 mm³ tumour fragments into female NU(NCr)-Foxn1nu mice (5–6 weeks old). Tumour volume was calculated as (length × width²)/2. Once tumours reached 150–200 mm³, mice were randomized to receive vehicle or vitamin C (4 g/kg, intraperitoneally, daily). All animal experiments were conducted in accordance with institutional guidelines and approved by the relevant ethics committees (PROEXP142-17; PIC124-21_FJD).

### Statistical analysis

Statistical analyses were performed using GraphPad Prism 6. Data are presented as mean ± SD or SEM as indicated. Comparisons between two groups were performed using unpaired t-tests with Welch’s correction. Multiple-group comparisons were analyzed using one-way ANOVA followed by Tukey’s post hoc test. Tumour growth data were analyzed using Kruskal–Wallis tests with pairwise Wilcoxon comparisons. P < 0.05 was considered statistically significant.

## Results

### ACLY activity regulates MDR1 expression in colorectal cancer

**Fig. 1:**
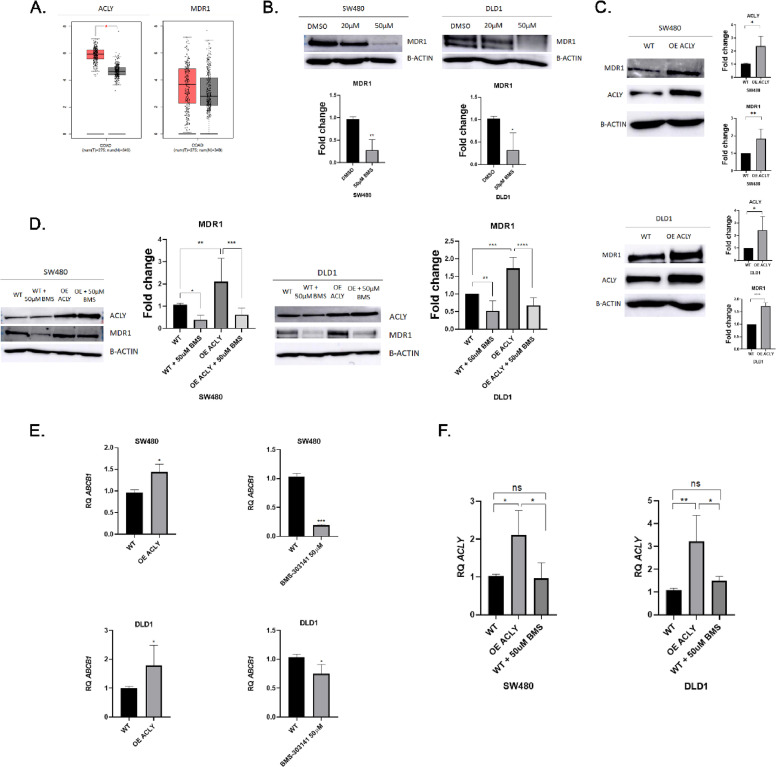
**ACLY activity regulates MDR1 expression in colorectal cancer.** (A) Transcript levels of ACLY and MDR1 (ABCB1) in colorectal cancer (red) and normal colon tissues (grey) analyzed using GEPIA (TCGA/GTEx datasets). (B) Immunoblot analysis of MDR1 in SW480 and DLD1 cells treated with the ACLY inhibitor BMS-303141 (20 or 50 μM, 48 h). (C) Immunoblot analysis of ACLY and MDR1 in SW480 and DLD1 cells transduced with empty vector (pLV) or ACLY-overexpressing vector (pLV[Exp]-hACLY). (D) Immunoblot analysis of ACLY and MDR1 in control and ACLY-overexpressing cells treated with BMS-303141 (50 μM, 48 h). (E) Relative ABCB1 mRNA levels in control and ACLY-overexpressing cells, and in cells treated with BMS-303141. (F) Relative ACLY mRNA levels under the same conditions. Data are presented as mean ± SD (n = 3 unless otherwise indicated). Statistical significance was determined using unpaired two-tailed t-tests or one-way ANOVA with Tukey’s post hoc test. *P < 0.05; **P < 0.01; ***P < 0.001.

To examine the relationship between ACLY and MDR1 in colorectal cancer (CRC), transcript levels were first analyzed in patient-derived datasets using GEPIA (TCGA/GTEx). ACLY expression was significantly elevated in tumour tissue (red) compared with normal colon mucosa (grey), whereas MDR1 (ABCB1) expression showed greater inter-sample variability (Fig. 1A).

To determine whether ACLY activity influences MDR1 expression, CRC cell lines were treated with the ACLY inhibitor BMS-303141. Pharmacological inhibition of ACLY resulted in a marked, dose-dependent reduction in MDR1 protein levels in both SW480 and DLD1 cells, exceeding 50% at 50 μM, without affecting total ACLY protein abundance (Fig. 1B & 1D). These findings indicate that MDR1 expression is sensitive to ACLY enzymatic activity rather than protein levels per se.

To further assess the role of ACLY expression, stable ACLY-overexpressing models were generated. Enforced ACLY expression increased MDR1 protein levels in both cell lines (Fig. 1C), whereas treatment with BMS-303141 reversed this effect (Fig. 1D), supporting a requirement for ACLY catalytic activity in sustaining MDR1 expression.

At the transcriptional level, ACLY overexpression increased ABCB1 mRNA abundance, while ACLY inhibition reduced ABCB1 transcript levels (Fig. 1E). ACLY mRNA levels confirmed effective overexpression and pharmacological targeting (Fig. 1F).

Together, these results support a role for ACLY activity in regulating MDR1 expression, consistent with a transcriptional mechanism.

Given the established role of MDR1 in drug efflux and chemoresistance, we next assessed whether ACLY modulation impacts functional drug response. ACLY overexpression significantly increased resistance to 5-FU, as reflected by an approximately twofold increase in IC50 values (Supplementary Fig. S2).

### ACLY modulates histone acetylation and is associated with changes in MDR1 expression

ACLY has been reported to localize to the nucleus, where it contributes to the generation of acetyl-CoA required for histone acetylation. Given the role of histone acetylation in promoting chromatin accessibility and transcriptional activation, we investigated whether MDR1 expression is regulated through this mechanism.

**Fig. 2:**
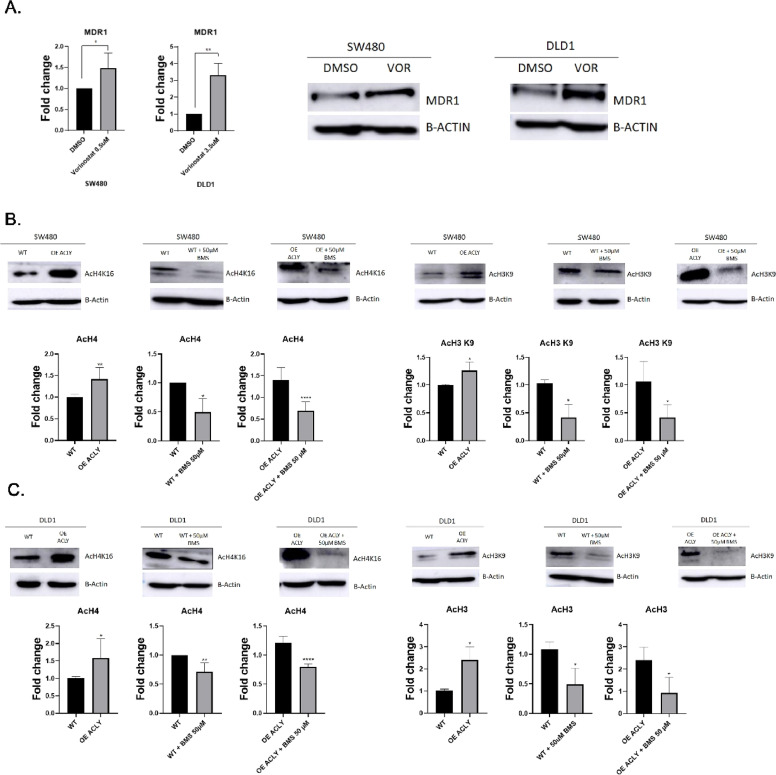
**ACLY activity modulates histone acetylation and MDR1 expression.** (A) Immunoblot analysis of MDR1 in SW480 and DLD1 cells treated with the histone deacetylase inhibitor vorinostat (VOR; 0.5 μM for SW480 and 3.5 μM for DLD1) or DMSO for 24 h. Representative blots and densitometric quantification relative to control are shown. (B) Immunoblot analysis of acetylated histone H3 (H3K9ac) and histone H4 (H4K16ac) in SW480 wild-type (WT) and ACLY-overexpressing (OE) cells treated with vehicle or the ACLY inhibitor BMS-303141 (50 μM, 48 h). (C) Immunoblot analysis of H3K9ac and H4K16ac in DLD1 cells under the same conditions. β-actin was used as a loading control. Data are presented as mean ± SD (n = 3). Statistical significance was determined using unpaired two-tailed t-tests. *P < 0.05; **P < 0.01; ***P < 0.001.

To assess the contribution of histone acetylation, SW480 and DLD1 cells were treated with the histone deacetylase inhibitor vorinostat (VOR). VOR treatment increased MDR1 protein levels in both cell lines (Fig. 2A), supporting the notion that MDR1 expression is sensitive to changes in histone acetylation.

We next examined histone acetylation at H3K9 and H4K16. ACLY overexpression increased acetylation at both marks, whereas pharmacological inhibition of ACLY with BMS-303141 reduced acetylation levels (Fig. 2B–C). These changes were consistent with the modulation of MDR1 expression observed under the same experimental conditions.

Together, these results indicate that ACLY activity influences histone acetylation and support a role for acetyl-CoA–dependent chromatin regulation in the control of MDR1 expression in CRC cells. While these findings are consistent with a transcriptional mechanism, locus-specific chromatin regulation at the MDR1 promoter was not directly assessed.

### ACLY expression is associated with resistance-related transcriptional programs in colorectal cancer

**Fig. 3:**
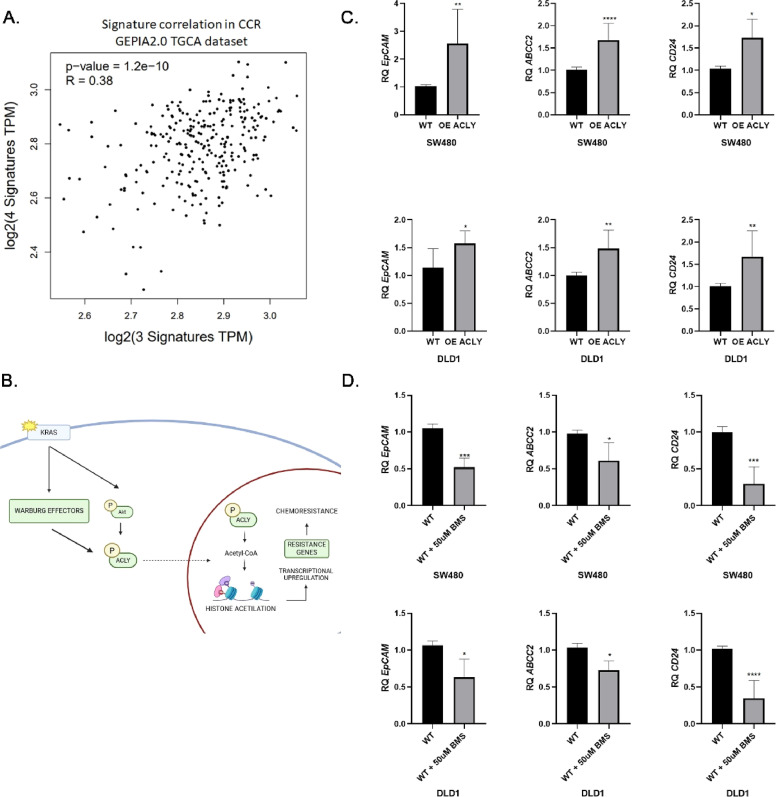
**ACLY expression is associated with resistance-related transcriptional programs in colorectal cancer.** (A) Correlation analysis between ACLY expression and a gene set associated with lipid metabolism (ACLY, ACSS2, ACSS1, FASN, SREBP1) and drug transport pathways (ABCB1, ABCC2, ABCG5, EpCAM, CD24) in colorectal cancer samples using GEPIA2 (TCGA dataset). (B) Schematic representation of a proposed model linking ACLY-dependent acetyl-CoA production to histone acetylation and transcriptional regulation in CRC cells. (C) Relative mRNA expression of EpCAM, ABCC2, and CD24 in SW480 and DLD1 cells overexpressing ACLY compared with empty vector controls. (D) Relative mRNA expression of EpCAM, ABCC2, and CD24 in SW480 and DLD1 cells treated with the ACLY inhibitor BMS-303141 (50 μM) compared with vehicle-treated controls. Gene expression levels were determined by qPCR and normalized to ACTB. Data are presented as mean ± SEM (n = 3). Statistical significance was determined using unpaired two-tailed t-tests. *P < 0.05; **P < 0.01; ***P < 0.001.

To explore the relationship between ACLY and broader transcriptional programs in colorectal cancer (CRC), correlation analysis was performed using TCGA transcriptomic data through the GEPIA2 platform. ACLY expression showed a positive association with a gene set enriched for lipid metabolism and drug transport pathways (R = 0.38; p < 1.2 × 10⁻¹⁰) (Fig. 3A). This gene set included transporters and markers previously linked to multidrug resistance and epithelial plasticity

To assess whether ACLY modulates components of these programs, we examined the expression of selected genes associated with drug transport and tumour cell phenotypes in CRC cell lines. ACLY overexpression in SW480 and DLD1 cells increased mRNA levels of ABCC2, EpCAM, and CD24, whereas pharmacological inhibition of ACLY with BMS-303141 reduced their expression (Fig. 3C,D). These changes were consistent with the regulation of MDR1 observed in the same models.

Together, these findings support an association between ACLY activity and transcriptional programs linked to drug transport and tumour cell state. While these data suggest a broader regulatory role for ACLY beyond MDR1, comprehensive characterization of ACLY-dependent transcriptional networks will require unbiased transcriptomic analyses.

### ACLY expression correlates with MDR1 levels and is enriched in advanced colorectal cancer

To assess the clinical relevance of the ACLY–MDR1 association, colorectal cancer (CRC) tissue microarrays comprising stage II (n = 307) and stage IV (n = 112) tumour samples were analyzed. Protein expression of ACLY and MDR1 was evaluated by immunohistochemistry and quantified using H-score assessment by a pathologist.

**Fig. 4:**
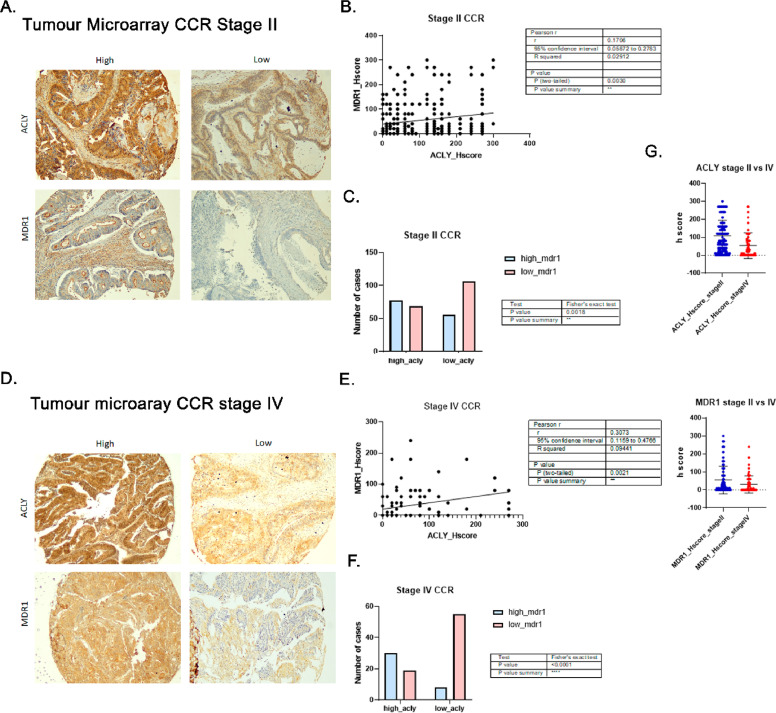
**Association between ACLY and MDR1 expression across colorectal cancer stages.** (A) Representative immunohistochemistry (IHC) images showing high and low ACLY and MDR1 staining in stage II colorectal cancer (CRC) specimens. (B) Pearson correlation analysis of ACLY and MDR1 H-scores in stage II tumours(r = 0.174; p = 0.003). (C) Distribution of ACLY and MDR1 expression categories in stage II CRC samples (Fisher’s exact test, p = 0.0018). (D) Representative IHC images of ACLY and MDR1 expression in stage IV CRC specimens. (E) Pearson correlation analysis of ACLY and MDR1 H-scores in stage IV tumours (r = 0.307; p = 0.002). (F) Distribution of ACLY and MDR1 expression categories in stage IV CRC samples (Fisher’s exact test, p = 0.0001). (G) Comparison of ACLY and MDR1 H-scores between stage II and stage IV tumours. Data are presented as mean ± SD. Statistical significance was determined as indicated; *P < 0.05; **P < 0.01; ***P < 0.001.

In stage II tumours, ACLY expression showed a modest but significant positive correlation with MDR1 levels (Pearson r = 0.171, p = 0.003; Fisher’s exact test p = 0.0018) (Fig. 4A–C). In stage IV tumours, this association was strengthened (Pearson r = 0.307, p < 0.0001; Fisher’s exact test p < 0.0001) (Fig. 4D–F). In addition, both ACLY and MDR1 expression levels were significantly increased in stage IV compared with stage II samples.

These findings indicate that ACLY and MDR1 expression are associated in CRC and that this relationship becomes more pronounced in advanced disease. While correlative, the increased concordance observed in stage IV tumours is consistent with a potential contribution of ACLY-associated transcriptional programs to tumour progression and drug resistance. Further studies integrating clinical outcomes will be required to determine the prognostic or predictive value of this association.

### Vitamin C induces coordinated metabolic and chromatin-associated changes in colorectal cancer cells

To assess whether metabolic perturbation impacts ACLY-associated regulatory networks, SW480 and DLD1 cells were treated with vitamin C (5 mM, 4 h) and subjected to label-free quantitative proteomic analysis (LC–MS/MS, diaPASEF). This approach enabled unbiased profiling of protein abundance changes following metabolic disruption.

Proteomic analysis revealed significant modulation of proteins involved in chromatin organization and metabolic pathways. In particular, components of ATP-dependent chromatin remodeling complexes and Polycomb group proteins were differentially regulated following vitamin C treatment. In parallel, multiple metabolic pathways, including sulfur metabolism, propanoate metabolism, and nucleotide biosynthesis, were altered, indicating coordinated changes across metabolic and chromatin-associated processes.

**Fig. 5:**
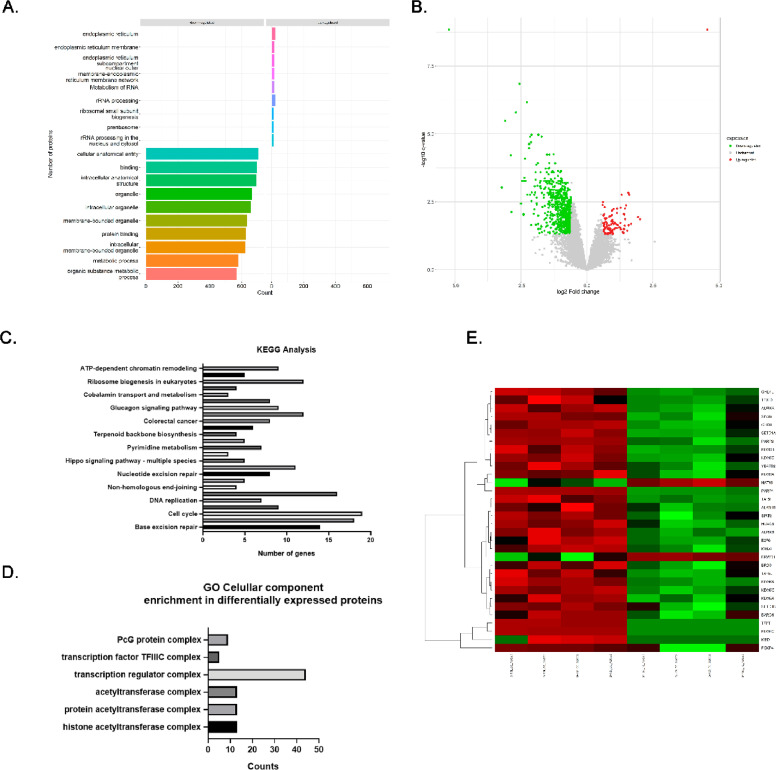
**Vitamin C induces coordinated changes in metabolic and chromatin-associated pathways in colorectal cancer cells.**(A) Gene Ontology (GO) enrichment analysis of proteins differentially expressed following vitamin C treatment (5 mM, 4 h). (B) Volcano plot showing significantly upregulated and downregulated proteins (log₂ fold change > 1, p < 0.05). (C) KEGG pathway enrichment analysis highlighting pathways related to chromatin organization, DNA replication, nucleotide metabolism, and cell cycle regulation. (D) GO Cellular Component analysis showing enrichment of chromatin-associated complexes, including transcription regulator complexes, histone acetyltransferase-containing complexes, and Polycomb group (PcG) assemblies. (E) Heatmap representation of differentially expressed chromatin-associated proteins in control and vitamin C-treated cells. Proteomic analysis was performed in SW480 and DLD1 cells using label-free LC–MS/MS (diaPASEF). Data represent combined analysis of both cell lines.

Gene Ontology enrichment analysis identified significant regulation of chromatin-associated complexes, including histone acetyltransferase-containing assemblies and transcriptional regulators (Fig. 5D). Consistently, heatmap analysis of epigenetic regulators showed a general decrease in the abundance of chromatin-modifying proteins upon vitamin C treatment (Fig. 5E).

These data indicate that vitamin C induces coordinated changes in metabolic and chromatin-associated pathways in CRC cells. While the effects of vitamin C are likely multifactorial, the observed modulation of chromatin regulatory complexes is consistent with disruption of acetyl-CoA–dependent processes linked to ACLY activity.

### Vitamin C modulates ACLY-associated metabolic and epigenetic regulation of MDR1 expression

**Fig. 6:**
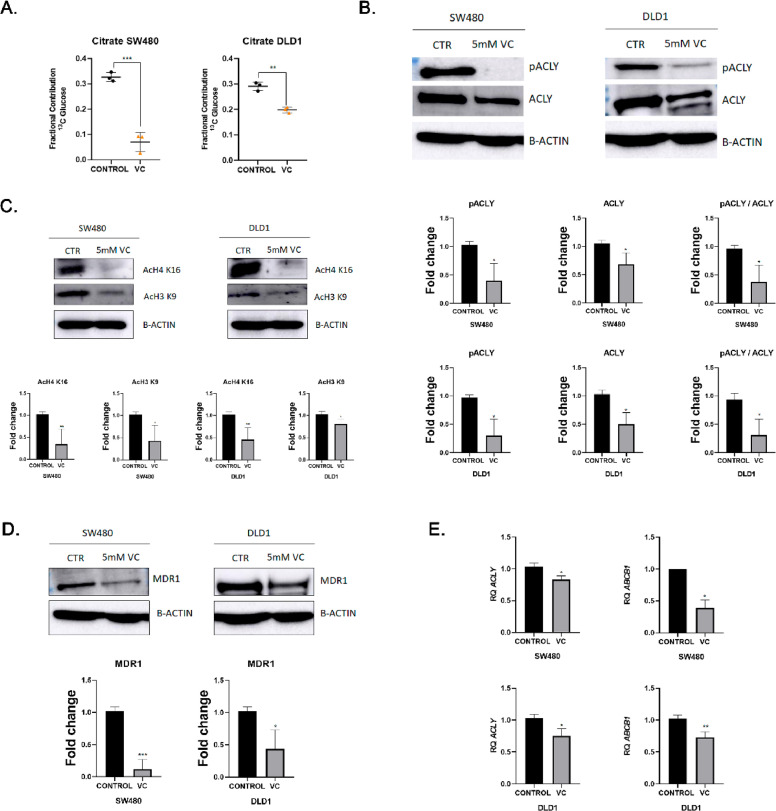
**Metabolic and epigenetic consequences of vitamin C treatment in colorectal cancer cells.**(A) Quantification of ¹³C-glucose-derived citrate in SW480 and DLD1 cells treated with vitamin C (5 mM) for 4 h (n = 3). (B) Immunoblot analysis of total ACLY and phosphorylated ACLY at Ser455 following vitamin C treatment (5 mM) (n = 3). (C) Immunoblot analysis and quantification of acetylated histone H4 (AcH4K16) and histone H3 (AcH3K9) in SW480 and DLD1 cells after vitamin C exposure (n = 3). (D) MDR1 (ABCB1) protein levels in SW480 and DLD1 cells treated with vitamin C (5 mM), quantified relative to vehicle control (n = 3). (E) Relative ACLY and ABCB1 mRNA expression determined by qPCR after 6 h of vitamin C treatment (5 mM) in SW480 and DLD1 cells (n = 3). Data are presented as mean ± SEM. Statistical significance was determined using unpaired two-tailed t-tests. *P < 0.05; **P < 0.01; ***P < 0.001.

Based on the observed proteomic changes in chromatin-associated pathways, we next examined whether metabolic perturbation by vitamin C impacts ACLY-dependent regulation. SW480 and DLD1 cells were subjected to 13C-glucose tracing following treatment with vitamin C (5 mM). Metabolic profiling revealed a reduction in 13C-labelled citrate, the immediate substrate of ACLY, indicating impaired citrate flux (Fig. 6A).

Consistent with these metabolic changes, vitamin C treatment reduced both total ACLY protein levels and its phosphorylated form (pS455-ACLY) (Fig. 6B). Given the role of ACLY in acetyl-CoA production, we assessed histone acetylation status. Vitamin C exposure decreased acetylation of histone H3K9 and H4K16 in both CRC cell lines (Fig. 6C), consistent with reduced acetyl-CoA–dependent chromatin modification.

We then evaluated the impact on MDR1 expression. Vitamin C treatment reduced MDR1 protein levels, and quantitative PCR analysis confirmed a corresponding decrease in ABCB1 mRNA expression (Fig. 6D,E).

Together, these findings indicate that vitamin C alters metabolic flux and is associated with reduced ACLY activity, decreased histone acetylation, and downregulation of MDR1 expression. While vitamin C is known to exert pleiotropic effects, these data are consistent with modulation of ACLY-dependent metabolic–epigenetic regulation in CRC cells.

To assess whether redox-dependent mechanisms contribute to the observed effects of vitamin C, SW480 cells were treated with the antioxidant N-acetylcysteine (NAC). NAC pre-treatment partially attenuated the reduction in MDR1 protein levels induced by vitamin C (Supplementary Fig. S3), suggesting that reactive oxygen species (ROS) contribute to this response, although they do not fully account for the observed changes.

### Vitamin C reduces tumour growth and is associated with modulation of ACLY-linked pathways in KRAS-mutant colorectal cancer PDX models

**Fig. 7:**
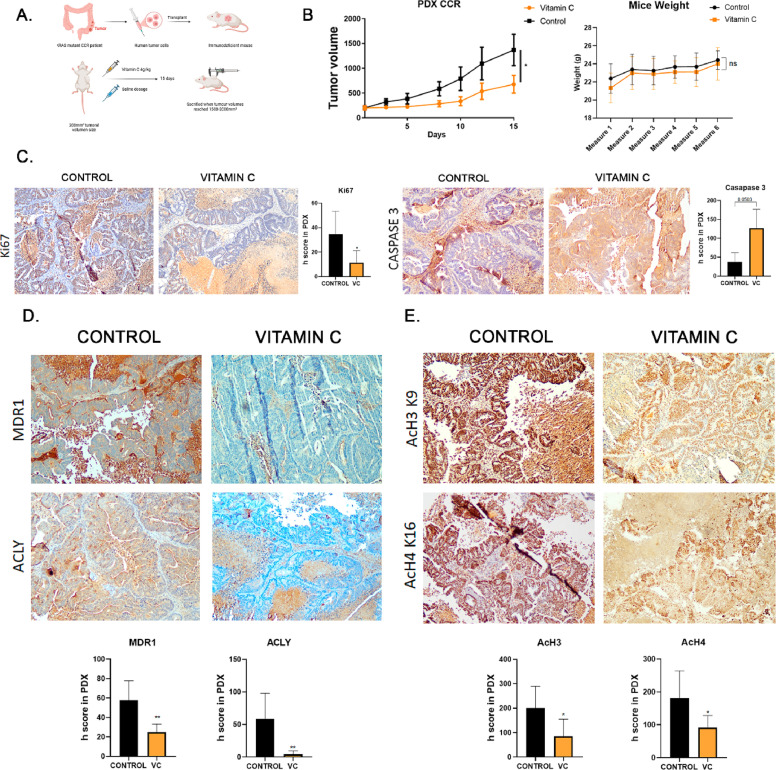
**Vitamin C reduces tumour growth and modulates ACLY-associated markers in KRAS-mutant colorectal cancer PDX models.** (A) Schematic representation of the treatment schedule. Tumour-bearing mice received vitamin C (4 g/kg, intraperitoneally) or saline daily. (B) Tumour growth curves and percentage of tumour growth inhibition in vitamin C- and saline-treated mice (n = 8 per group). Tumour size in control animals reached endpoint criteria during the study period. Mice weight in grams. (C) Representative Ki67 immunohistochemistry (IHC) images and Caspase 3 and quantification of Ki67-positive tumour cells are shown. (D) Representative IHC images and H-score quantification of MDR1 and ACLY expression in tumours from control and vitamin C-treated mice. (E) Representative IHC images and H-score quantification of histone acetylation marks H3K9ac and H4K16ac in tumour tissues. Data are presented as mean ± SEM (n = 8). Statistical significance was determined using unpaired two-tailed t-tests. *P < 0.05; **P < 0.01; ***P < 0.001.

To evaluate the *in vivo* relevance of ACLY-associated regulatory mechanisms, the effects of pharmacological vitamin C were assessed in KRAS-mutant colorectal cancer patient-derived xenograft (PDX) models. Vitamin C treatment significantly reduced tumour growth compared with vehicle-treated controls over the course of the experiment (Fig. 7B). Tumour growth in control animals reached endpoint criteria, requiring termination of the experiment, whereas treated tumours remained significantly smaller. A decrease in proliferative activity was observed, as indicated by reduced Ki67 staining.

In addition to effects on tumour growth, vitamin C treatment was associated with reduced protein expression of ACLY and MDR1 in tumour tissues (Fig. 7C). Histological analysis further showed decreased acetylation of histone marks H3K9 and H4K16 in treated tumours (Fig. 7D), consistent with altered chromatin-associated regulation *in vivo*.

Together, these findings indicate that vitamin C treatment impacts tumour growth and is associated with modulation of ACLY expression, histone acetylation, and MDR1 levels in KRAS-mutant CRC models. While vitamin C exerts pleiotropic effects, these data support the *in vivo* relevance of ACLY-associated metabolic–epigenetic regulation.

## Discussion

In this study, we identify ATP-citrate lyase (ACLY) as a metabolic regulator associated with MDR1 expression in colorectal cancer (CRC). Our data support a model in which ACLY activity contributes to the maintenance of histone acetylation states linked to transcriptional regulation of MDR1 and related gene programs. By integrating pharmacological and genetic approaches with metabolic and *in vivo* analyses, our findings position ACLY at the interface between metabolic flux and chromatin-associated regulation in CRC.

ACLY is a key source of nucleocytosolic acetyl-CoA, linking nutrient availability to histone acetylation [[31], [32]] and transcriptional control [[33], [34], [35], [36]]. In this context, our data showing concordant changes in ACLY activity, histone acetylation (H3K9ac and H4K16ac), and MDR1 expression are consistent with a role for acetyl-CoA-dependent chromatin regulation in efflux-associated transcriptional programs[[35], [36]]. While these findings support a transcriptional mechanism, locus-specific regulation at the MDR1 promoter was not directly assessed, and additional studies will be required to define the chromatin landscape governing MDR1 expression.

Metabolic tracing experiments further indicate that perturbation of citrate availability impacts ACLY-associated pathways. Vitamin C treatment reduced incorporation of glucose-derived carbon into citrate, consistent with altered metabolic flux, and was associated with decreased ACLY abundance and phosphorylation [37]. These changes were accompanied by reduced histone acetylation and MDR1 expression, supporting a link between metabolic state and chromatin-associated regulation [[38], [39]]. Notably, the partial attenuation of MDR1 downregulation by NAC suggests that redox-dependent mechanisms contribute to the effects of vitamin C. However, given the pleiotropic nature of vitamin C, including its roles in redox biology and epigenetic regulation, these effects cannot be attributed exclusively to ACLY modulation [[40], [41]].

The clinical data provide additional support for the relevance of this axis. ACLY and MDR1 expression were positively correlated in CRC specimens, with a stronger association observed in advanced-stage tumours. Although correlative, these findings are consistent with a model in which metabolic-epigenetic coupling becomes more prominent during tumour progression. Future studies integrating clinical outcomes will be required to determine whether ACLY/MDR1 co-expression has prognostic or predictive value.

Our proteomic analyses suggest that metabolic perturbation is associated with broader changes in chromatin-associated complexes, including histone acetyltransferase-containing assemblies and Polycomb-related proteins. These observations are consistent with emerging evidence linking metabolic enzymes to epigenetic regulation in cancer [[42], [43], [44], [45], [46], [47], [48]]. However, comprehensive characterization of ACLY-dependent transcriptional networks will require unbiased transcriptomic and chromatin-based approaches.

*In vivo* data further support the translational relevance of these findings. In KRAS-mutant PDX models, vitamin C treatment reduced tumour growth and was associated with decreased ACLY expression, histone acetylation, and MDR1 levels. Importantly, tumour growth in control animals reached endpoint criteria during the experimental period, supporting the biological significance of the observed differences. While these data indicate that modulation of metabolic pathways impacts tumourbehaviour, the contribution of ACLY-specific mechanisms relative to other effects of vitamin C remains to be determined.

Although preliminary functional data (Supplementary Fig. S2) support a role for ACLY in modulating sensitivity to 5-FU, more comprehensive analyses will be required to fully define its contribution to chemoresistance. In addition, locus-specific chromatin regulation at the MDR1 promoter was not directly assessed. Finally, the pleiotropic nature of vitamin C limits mechanistic attribution to a single pathway.

Despite these limitations, our findings define a metabolic-epigenetic framework in which ACLY is associated with transcriptional regulation of MDR1 and related programs in CRC. Targeting metabolic dependencies that influence chromatin state may represent a strategy to modulate efflux-associated resistance mechanisms. Further studies will be required to establish the therapeutic potential of this approach and to define the molecular determinants that govern ACLY-dependent regulation in colorectal cancer.

## Conclusion

Our study identifies ACLY as a metabolic regulator associated with histone acetylation and MDR1 expression in colorectal cancer. The data support a model in which acetyl-CoA availability links metabolic state to chromatin-associated regulation of efflux-related gene expression.

Perturbation of citrate-dependent metabolic pathways, including vitamin C treatment, was associated with coordinated changes in ACLY levels, histone acetylation, and MDR1 expression *in vitro* and in KRAS-mutant PDX models. While these effects are consistent with modulation of ACLY-associated processes, the pleiotropic nature of vitamin C and the absence of locus-specific analyses warrant further investigation.

Overall, these findings highlight the potential role of metabolic-chromatin coupling in the regulation of drug resistance programs in CRC and suggest that targeting metabolic dependencies may represent a strategy to modulate efflux-associated resistance.

## Abbreviations

ABCB1: ATP-binding cassette subfamily B member 1, ABC: ATP-binding cassette, ACLY: ATP-citrate lyase, CRC: colorectal cancer, EGFR: epidermal growth factor receptor, GO: Gene Ontology, GTEx: Genotype-Tissue Expression, H3K9: histone H3 lysine 9, H4K16: histone H4 lysine 16, HDAC: histone deacetylase, IHC: immunohistochemistry, KEGG: Kyoto Encyclopedia of Genes and Genomes, KRAS: Kirsten rat sarcoma viral oncogene homolog, LC–MS/MS: liquid chromatography–tandem mass spectrometry, MDR: multidrug resistance, MDR1: multidrug resistance protein 1, PDX: patient-derived xenograft, PROTAC: proteolysis-targeting chimera, TCGA: The Cancer Genome Atlas, VOR: vorinostat.

## CRediT authorship contribution statement

**Ana García-Bautista:** Writing – original draft, Methodology, Investigation, Data curation. **Aiora Cenigaonandia-Campillo:** Methodology, Investigation. **Anxo Rio-Vilariño:** Validation, Software, Data curation. **Lara Sanz-Criado:** Resources, Investigation. **Marta Selva-Giménez:** Validation, Investigation. **Raquel Perez-Antolín:** Methodology, Investigation. **Arancha Cebrián:** Resources, Methodology, Investigation. **Laura García-García:** Project administration, Investigation. **María Jesús Fernández-Aceñero:** Methodology, Formal analysis, Data curation. **Natalia Baños-Herraiz:** Visualization, Validation, Investigation, Formal analysis. **Lorena Mozas-Vivar:** Resources, Formal analysis, Data curation. **Estrella Núñez-Delicado:** Supervision, Resources, Funding acquisition. **Jesús García-Foncillas:** Writing – original draft, Supervision, Resources, Funding acquisition. **Óscar Aguilera:** Writing – review & editing, Supervision, Funding acquisition, Conceptualization.

## Declaration of competing interest

The authors declare that they have no known competing financial interests or personal relationships that could have appeared to influence the work reported in this paper.

## Appendix Supplementary materials

Image, application 1

## References

[1] Kekelidze M., D’Errico L., Pansini M., Tyndall A., Hohmann J. (2013). Colorectal cancer: current imaging methods and future perspectives for the diagnosis, staging and therapeutic response evaluation. World J. Gastroenterol..

[2] Krauß D. (2025). EGFR controls transcriptional and metabolic rewiring in KRASG12D colorectal cancer. EMBo Mol. Med..

[3] Imamura Y. (2014). Analyses of clinicopathological, molecular, and prognostic associations of KRAS codon 61 and codon 146 mutations in colorectal cancer: cohort study and literature review. Mol. Cancer.

[4] Fojo T. (2024). Sotorasib in KRAS-mutated colorectal cancer. N. Engl. J. Med..

[5] Hallin J. (2020). The KRASG12C inhibitor MRTX849 provides insight toward therapeutic susceptibility of KRAS-mutant cancers in mouse models and patients. Cancer Discov..

[6] Popow J. (2024). Targeting cancer with small-molecule pan-KRAS degraders. Science.

[7] Kurimchak A.M. (2022). The drug efflux pump MDR1 promotes intrinsic and acquired resistance to PROTACs in cancer cells. Sci. Signal..

[8] de Groot R.A., Reedijk D., Faucher Q., Mihăilă S.M., Masereeuw R. (2025). Strategies for overcoming ABC transporter-mediated multidrug resistance in colorectal cancer. Am. J. Physiol. Cell Physiol..

[9] Hu T., Li Z., Gao C.Y., Cho C.H. (2016). Mechanisms of drug resistance in colon cancer and its therapeutic strategies. World J. Gastroenterol..

[10] Robey R.W. (2018). Revisiting the role of ABC transporters in multidrug-resistant cancer. Nat. Rev. Cancer.

[11] Dahlmann M. (2020). Restoring treatment response in colorectal cancer cells by targeting MACC1-dependent ABCB1 expression in combination therapy. Front. Oncol..

[12] Tian Y. (2023). Mechanism of multidrug resistance to chemotherapy mediated by P-glycoprotein. Int. J. Oncol..

[13] Lei Z.N. (2024). ABCB1-dependent collateral sensitivity of multidrug-resistant colorectal cancer cells to the survivin inhibitor MX106-4C. Drug Resist. Updat..

[14] Zhang Y. (2020). Poziotinib inhibits the efflux activity of the ABCB1 and ABCG2 transporters and the expression of the ABCG2 transporter protein in multidrug resistant colon cancer cells. Cancers.

[15] Eckford P.D.W., Sharom F.J. (2006). P-glycoprotein (ABCB1) interacts directly with lipid-based anti-cancer drugs and platelet-activating factors. Biochem. Cell Biol..

[16] Troost J., Lindenmaier H., Haefeli W.E., Weiss J. (2004). Modulation of cellular cholesterol alters P-glycoprotein activity in multidrug-resistant cells. Mol. Pharmacol..

[17] Hegedüs C., Telbisz Á., Hegedűs T., Sarkadi B., Özvegy-Laczka C. (2015). Lipid regulation of the ABCB1 and ABCG2 multidrug transporters. Adv. Cancer Res..

[18] Zhou Y. (2013). ATP citrate lyase mediates resistance of colorectal cancer cells to SN38. Mol. Cancer Ther..

[19] Granchi C. (2018). ATP citrate lyase (ACLY) inhibitors: an anti-cancer strategy at the crossroads of glucose and lipid metabolism. Eur. J. Med. Chem..

[20] Wellen K.E. (2009). ATP-citrate lyase links cellular metabolism to histone acetylation. Science.

[21] Zheng W., Tasselli L., Li T., Chua K.F. (2021). Mammalian SIRT6 represses invasive cancer cell phenotypes through ATP citrate lyase (ACLY)-dependent histone acetylation. Genes.

[22] Sivanand S. (2017). Nuclear acetyl-CoA production by ACLY promotes homologous recombination. Mol. Cell.

[23] Liu Y., Jiang X., Jing D., Lin Y., Gao R., Zhao Q. (2025). ACSL4 knockdown inhibits colorectal cancer progression through stimulating anti-tumor immunity. Neoplasia.

[24] Cenigaonandia-Campillo A. (2021). Vitamin C activates pyruvate dehydrogenase (PDH) targeting the mitochondrial tricarboxylic acid cycle in hypoxic KRAS mutant colon cancer. Theranostics.

[25] Adant I. (2022). Pyruvate and uridine rescue the metabolic profile of OXPHOS dysfunction. Mol. Metab..

[26] Demichev V., Messner C.B., Vernardis S.I., Lilley K.S., Ralser M. (2020). DIA-NN: neural networks and interference correction enable deep proteome coverage in high throughput. Nat. Methods.

[27] S. Camara-Fuentes, D. Gutierrez-Blazquez, M.L. Hernaez, C. Gil, TraianProt: a user-friendly R shiny application for wide format proteomics data downstream analysis, arXiv. 2024;2412.15806.

[28] Ritchie M.E. (2015). limma powers differential expression analyses for RNA-sequencing and microarray studies. Nucleic. Acids. Res..

[29] Yu G., Wang L.G., Han Y., He Q.Y. (2012). clusterProfiler: an R package for comparing biological themes among gene clusters. OMICS.

[30] Kanehisa M., Goto S. (2000). KEGG: kyoto encyclopedia of genes and genomes. Nucleic. Acids. Res..

[31] Lee J.V. (2014). Akt-dependent metabolic reprogramming regulates tumor cell histone acetylation. Cell Metab..

[32] Wang C., Ma X. (2025). The role of acetylation and deacetylation in cancer metabolism. Clin. Transl. Med..

[33] Linder S.J., Mostoslavsky R. (2017). Put your mark where your damage is: acetyl-CoA production by ACLY promotes DNA repair. Mol. Cell.

[34] Umemoto T. (2022). ATP citrate lyase controls hematopoietic stem cell fate and supports bone marrow regeneration. EMBO J..

[35] Morrison A.J. (2022). Cancer cell metabolism connects epigenetic modifications to transcriptional regulation. FEBS. J..

[36] Campbell S.L., Wellen K.E. (2018). Metabolic signaling to the nucleus in cancer. Mol. Cell.

[37] Wei J. (2019). An allosteric mechanism for potent inhibition of human ATP-citrate lyase. Nature.

[38] Dominguez M., Brüne B., Namgaladze D. (2021). Exploring the role of ATP-citrate lyase in the immune system. Front. Immunol..

[39] Russo M., Pileri F., Ghisletti S. (2023). Novel insights into the role of acetyl-CoA producing enzymes in epigenetic regulation. Front. Endocrinol..

[40] Khwairakpam A.D. (2015). ATP citrate lyase (ACLY): a promising target for cancer prevention and treatment. Curr. Drug Targets..

[41] Grobs Y. (2024). ATP citrate lyase drives vascular remodeling in systemic and pulmonary vascular diseases through metabolic and epigenetic changes. Sci. Transl. Med..

[42] Martínez-Reyes I., Chandel N.S. (2018). Acetyl-CoA-directed gene transcription in cancer cells. Genes Dev..

[43] Sivanand S., Viney I., Wellen K.E. (2018). Spatiotemporal control of acetyl-CoA metabolism in chromatin regulation. Trends. Biochem. Sci..

[44] Lee J.V. (2018). Acetyl-CoA promotes glioblastoma cell adhesion and migration through Ca2+-NFAT signaling. Genes Dev..

[45] Hoxhaj G., Manning B.D. (2020). The PI3K-AKT network at the interface of oncogenic signalling and cancer metabolism. Nat. Rev. Cancer.

[46] Snaebjornsson M.T., Schulze A. (2018). Non-canonical functions of enzymes facilitate cross-talk between cell metabolic and regulatory pathways. Exp. Mol. Med..

[47] He W., Li Q., Li X. (2023). Acetyl-CoA regulates lipid metabolism and histone acetylation modification in cancer. Biochim. Acta Rev. Cancer.

[48] da Silva-Diz V. (2025). A feedforward loop between ACLY and MYC supports T-ALL progression in vivo. Blood Neoplasia.

