# Supplementary material for: Metabolic regulation of histone acetylation by ACLY supports MDR1 expression in colorectal cancer and highlights a targetable vulnerability
Source: Neoplasia. 2026 Apr 30;77:101314. doi: 10.1016/j.neo.2026.101314 (PMC13142016; doi:10.1016/j.neo.2026.101314)
Supplement: Supplementary file 1 [file mmc1.docx]

Supplementary Figures


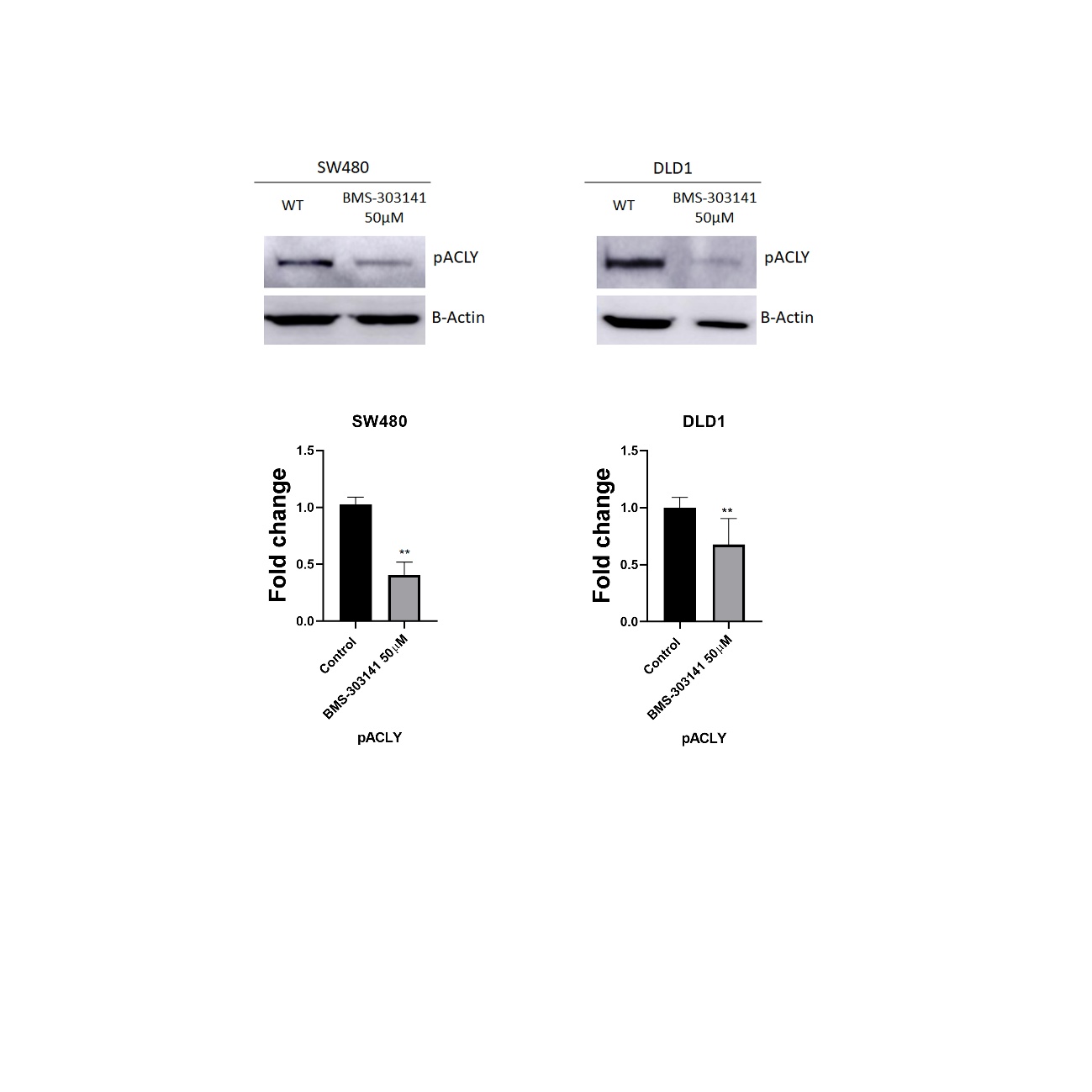


**Figure S1. BMS-303141 reduces ACLY phosphorylation at Ser455.** Immunoblot analysis of phospho ACLY in SW480 and DLD1 cells treated with the ACLY activity inhibitor (BMS-303141) Representative blots and densitometric quantification relative to control are shown. Fold change representation respect to B-Actin. Data are presented as mean ± SEM (n = 3). Statistical significance was determined using unpaired two-tailed t-tests. *P < 0.05; **P < 0.01; ***P < 0.001.


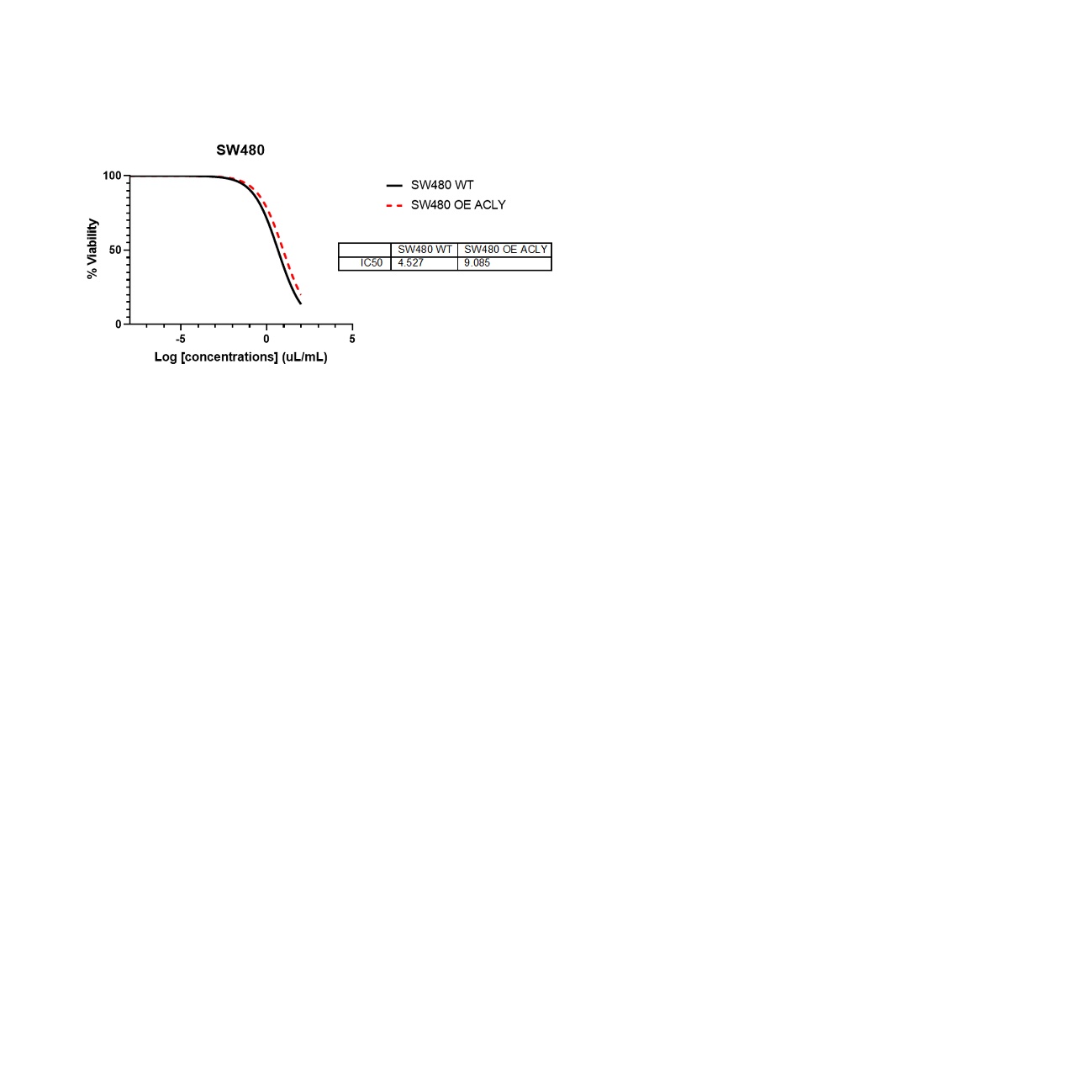


**Figure S2. ACLY overexpression confers more resistance to 5-fluorouracil**. Dose-response curves for wild type (WT) and ACLY-overexpressing (OE) cells treated with increasing concentrations of 5-fluorouracil. Data were fitted using nonlinear regression. ACLY overexpression induced a rightward shift in the dose-response curve and significantly increase IC50 values.


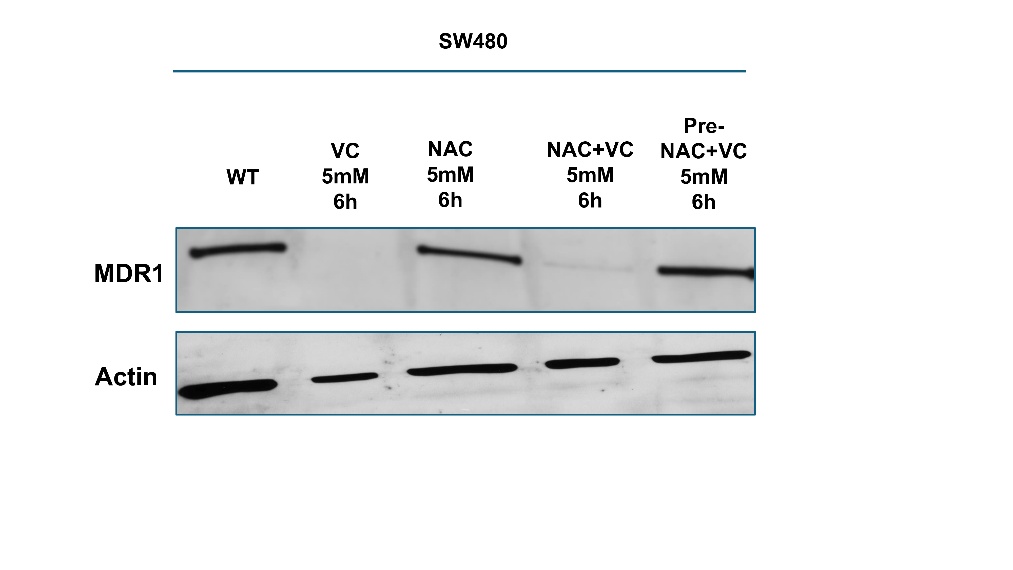


**Figure S3. Contribution of redox-dependent mechanisms to vitamin C-mediated modulation of MDR1 expression.** SW480 cells were treated with vitamin C (5 mM, 6 h), NAC (5 mM, 6 h), or their combination, including NAC pre-treatment. Representative immunoblot analysis of MDR1 is shown, with β-actin as loading control. NAC partially attenuated the reduction in MDR1 levels induced by vitamin C, suggesting a contribution of ROS-dependent mechanisms.
